# eCorsi: implementation and testing of the Corsi block-tapping task for digital tablets

**DOI:** 10.3389/fpsyg.2014.00939

**Published:** 2014-09-02

**Authors:** Riccardo Brunetti, Claudia Del Gatto, Franco Delogu

**Affiliations:** ^1^Department of Human Sciences, Università Europea di RomaRome, Italy; ^2^Department of Humanities, Social Sciences, and Communication, College of Arts and Sciences, Lawrence Technological UniversitySouthfield, MI, USA

**Keywords:** visuo-spatial working memory, computer-based neuropsychological testing, digitization, touchscreen

## Abstract

The Corsi block-tapping task is a widely used test to assess visuo-spatial working memory. The test is traditionally administered using nine square blocks positioned on a wooden board, but numerous digital versions have been developed. In this study, we tested one-hundred and seven participants divided into two age groups (18–30 and over 50) in forward, backward and supraspan-forward conditions with eCorsi, a tablet version of the Corsi task. Compared to the traditional physical board, eCorsi has several advantages, including: simple installation, set-up, and use; considerably increased accuracy in presentation timing, automatic measures of span and reaction times, in both the forward and backward response modalities. Results showed that average span and error rates were essentially analogous to the ones obtained in the main standardization studies, which have used the original physical version of the Corsi test. Furthermore, timing results provide new indications about the mechanisms underlying spatial sequence processing, suggesting that the subject's response is not planned during sequence presentation, but between the end of the presentation and the beginning of the response.

## Introduction

The Corsi block tapping task (CBTT; Corsi, 1972) has been described as the single most important nonverbal task in neuropsychological research (Berch et al., 1998). It is extensively used in diagnostics for the assessment of visuo-spatial working memory (VSWM), but it also involves spatial attention (Smyth and Scholey, 1994). Moreover, it is often used as part of test batteries in the diagnostics of several diseases such as the early detection of Alzheimer's disease (Carlesimo et al., 1994), Korsakoff's syndrome (Haxby et al., 1983), schizophrenia (Chey et al., 2002), and to support hypotheses about the localization of focal brain lesions (Milner, 1971; De Renzi et al., 1977).

CBTT has also seen widespread use in experimental research (see for example Smyth et al., 1988; Fischer, 2001; Bo et al., 2011) as a measure of VSWM.

CBTT is administered with many variants. The traditional version consists of nine cubical blocks positioned on a board. In this version, typically, the examiner taps the blocks starting with sequences of two cubes. The subject has to reproduce a given sequence by tapping the blocks in the same sequence she saw (“forward” task), or in backward order (e.g., starting with the last block tapped by the experimenter and ending with the first one; “backward” task). If a certain proportion of the sequences is reproduced correctly (usually 1/2, 2/3, or 3/5 of the trials per sequence length), the sequence length increases by one item. The procedure ends when the number of wrong reproductions exceeds the proportion of admissible errors per length. This classic version of the CBTT can have many structural variations in size, position of the blocks on the board, number of blocks (see Kessels et al., 2000 and Berch et al., 1998 for reviews). It has been noted that such differences in the physical layout can be the cause of a great deal of variations in scores between studies (Smirni et al., 1983; Kemps, 1999), along with age differences (Kessels et al., 2008). In addition to the physical versions of the CBTT, several computer based-forms of the test have been developed (Morris and Baddeley, 1988; Smyth and Scholey, 1994; Nelson et al., 2000; LeFevre et al., 2010). In these versions, instead of cubes to be tapped on a board, the setup consists of squares that flash on a computer screen. Participants reproduce the sequences either by tapping blocks on a (touch) screen (Smyth and Scholey, 1994; Vandierendonck et al., 2004) or by using a mouse to click on the blocks (Pearson and Sahraie, 2003; LeFevre et al., 2010). Finally, more peculiar versions of CBTT include a haptic Corsi (Ruggiero and Iachini, 2010) and even a “walking Corsi” (Piccardi et al., 2008). All these variations make it difficult to understand what scores we should expect from a given sample. In clinical neuropsychology this issue is particularly relevant, as it is crucial to have a reliable test with appropriate normative data for the assessment of spatial working memory. An attempt to standardize the scores of the CBTT has been made by Kessels et al. (2008). They concluded that CBTT in its forward version can be effectively used to assess visuo-spatial short-term memory in patients with brain damage and that it is selective for the side of the lesion (Kessels et al., 2000), while they questioned the clinical use of the backward version of the task to assess working memory deficits (Kessels et al., 2008).

As suggested by many studies (Berch et al., 1998; Fischer, 2001; Pagulayan et al., 2006; Rowe et al., 2008), in the community of clinicians and researchers there is a growing need for new standardized and automated ways of assessing cognitive abilities. Easy-to-manage diagnostic tools that make it possible to overcome the limitation of paper and pencil tests are more necessary than ever. CBTT is no exception: an easy to use, computerized version of the Corsi test would be very useful for clinicians and researchers. Nelson et al. (2000) investigated the potential differences between administering the CBTT manually or by means of an automated device. The automation, obtained by wiring a modified Corsi board to a computer, featured flashing lights to administer the sequences to the subjects and button-like devices on the top of blocks to record their responses. Their results confirm that there are no substantial differences between the two versions in terms of subjects' performance. However, while their study could have potentially opened the way to new research methods (e.g., including in the study a response timing analysis), their custom-made apparatus shared many features of the traditional CBTT (e.g., size, materials), making it essentially as bulky and even more complex than the traditional one.

Tablet computers could be a valid alternative to traditional methods. Diagnostic tools on tablets have several advantages compared to their traditional counterparts. They are light and handy, they have become extremely easy to purchase at a low price, they are user-friendly for all kinds of participants (including children), they provide automatic data collection (therefore minimizing human error) and identical conditions in stimuli presentation across subjects, they allow for a precise customization of stimuli presentation, and they allow the collection of additional data (e.g., reaction times, see Fischer, 2001). The introduction of a computer-based form of the CBTT for tablet computers seems to bring all of the above-mentioned advantages to research and clinical practices.

In this study we introduce eCorsi (Brunetti et al., 2013), a software developed for the administration of a computer-based version of the CBTT through tablets. The purpose of our study is multifold. First, we describe a number of advantages deriving from the use of this device instead of the traditional version of CBTT. Second, we provide a data set and compare our scores with scores from both traditional Corsi and other digitized versions. Third, we analyze the results obtained in light of the possible advantages that a computer-based version could offer to research and clinical diagnosis.

### Comparison with traditional CBTT versions

There are several analogies between traditional CBTT and eCorsi, like identical proportions, similar size of the blocks/squares, similar size of the board, possibility to customize the Inter-Stimulus and flash timing to equalize the timings of administration of eCorsi with any version of the CBTT. Moreover, the use of a touch interface instead of a mouse allows the motor programming involved to carry out the task to be essentially the same as in the traditional version.

Differences between traditional CBTT and eCorsi include a better control of the Inter-Stimulus presentation timings in the eCorsi. In fact, with manual tapping the temporal accuracy is particularly difficult to control by the examiner, who can (inadvertently) be slower or faster depending on several factors. Moreover, the examiner could change the finger used for tapping, the amplitude of hand and arm movements and the position of the limb in the intertapping intervals. Most studies do not even report the way in which the blocks were pointed at by the examiner. Moreover, neuropsychologists often complain that when administering particularly long sequences they are forced to slow down the pace of block tapping because they have to read the sequence in order to remember it (Haike Stralen, personal communication, 2012). The use of eCorsi can drastically reduce many sources of inter-trial and inter-test variability both between subjects and between examiners, while maintaining an extreme plasticity to be adapted to different research and clinical purposes through customization of timing intervals and its different task modes. Another obvious advantage is that all the trials are automatically recorded, and therefore checked for errors.

eCorsi seems particularly handy to test bedridden patients, a common condition in neuropsychological diagnostics. It could also be useful to test patients in operating theaters, for example when there is a need to determine residual spatial abilities after focal brain lesions. In fact, thanks to the monitor function, eCorsi allows for a remote administration of the test: not requiring examiner-patient proximity, eCorsi seems less invasive than traditional CBTT. We tested 107 participants in three different CBT tasks: the forward, the backward and the supraspan task (in the procedure described in Trojano et al., 1994), which will be described in detail in the Procedure.

## Methods

### Participants

One hundred and seven participants (58 female) with a mean age of 32.3 years (*SD* = 16.9) participated in the study. The sample was divided into two age groups, 73 young adults (average age 21.6 years, *SD* = 1.7; 42 female) and 34 elderly adults (average age 57.6 years, *SD* = 1.7; 13 female). Participants had a mean educational level of 14.82 years of schooling (range 8–18, *SD* = 2.09)^1^. The criterion for the inclusion in the elderly adults group was to be older than 50. All participants were right-handed and had normal or corrected-to-normal vision. None of the elderly participants reported any kind of neuropsychological disease when administered with a short anamnestic interview. Moreover, all participants were tested with the Short Portable Mental Status Questionnaire (SPMSQ; Pfeiffer, 1975) and none of them scored higher than 2, hence showing a normal intellectual functioning.

### Apparatus

eCorsi runs on a system made of two devices: a computer and a tablet. Though its implementation makes it cross-platform (Mac OS and Windows systems, iOS 6 and Android), here we will limit our discussion to the platforms we developed it on, a third generation Apple iPad 2 and a MacBook Pro laptop.

The two devices are wirelessly connected and use the User Datagram Protocol (UDP) to remotely send and receive Open Sound Control (OSC) messages. Compared to a wired connection, the average lag due to the wireless connection is, in optimal conditions (e.g., dedicated wi-fi, no other programs running on iPad and laptop, no internet connection on both devices, etc.), about 15 ms. Each device runs a different program. The tablet runs “Touch OSC,” a program by hexler, developed for iOS and Android, with a custom layout reproducing the Corsi board (see Supplementary Material for more details about the adaptation for the iPad screen). The laptop runs a patch developed in Max 6 (a graphical programming environment created by Miller Puckette, developed by David Zicarelli and distributed by Cycling'74) controlling sequence administration^2^. The participant sits in front of the tablet, while the experimenter/clinician sits in front of the computer. Basically, the whole test is managed by the computer program, which generates the sequences and records the data, while the tablet serves as a remote touchscreen. The trials are visualized on the tablet screen as a sequence of flashing squares and, when the sequence ends, the participant reproduces the sequence by tapping the squares which just flashed on the touchscreen. An icon on the screen signals the end of the sequence (a little blue circle appears when the last square is lit). The computer program features a “monitor,” namely a small tablet reproduction that shows at any moment what is displayed and tapped on the tablet's screen.

The program can work in three different modes: Span Sequence Mode, Free Trial Mode and Manual Mode.

Span Sequence Mode: this mode generates a sequence of progressive trials to assess the participant's span. The user (experimenter/clinician) can decide the number of trials (1–5) per sequence length and the number of correct responses necessary to progress to longer sequences. The standardized sequences used in Kessels et al. (2000, 2008) are implemented in the default span mode when 2 trials per sequence length is selected. When more trials per length are chosen, additional sequences are used which follows similar principles as in Kessels et al. (2000, 2008).

Free Trial Mode: the user can decide the length of the sequence (1–12 items) that will be then randomly generated by the program, thus avoiding block repetitions in sequences up to a length of 9 items.

Manual Mode: using this mode the user can create custom sequences on the “monitor” reproducing the tablet's screen by clicking on the blocks in the desired order. The program then visualizes the sequences on the tablet with controlled timing, before recording the participant's response.

eCorsi records all of the participant's responses and cumulative reaction times, along with correct sequences (either the fixed span sequences or the custom created sequences in free or manual modes) and errors. All of this data is then organized in a tab-separated text log file, along with personal information from the participant, such as age, gender, and number.

The program is fully and easily customizable in Inter-Stimulus Intervals (ISI) and block flash timing with millisecond precision, forward or backward response modality (namely if the participant has to reproduce the sequence in the same or in a reverse serial order), tablet on-screen error feedback for the participant, maximum admissible mistakes, and block sequences used to assess the VSWM span.

### Procedure

The participant sat on a chair in front of a table in which the tablet was horizontally placed on the table. On the screen, a traditional Corsi board structure showed 9 yellow square frames on a black background (please see Appendix A in Supplementary Material for exact layout and size). Each participant was tested in both forward and backward conditions, counterbalancing the order between subjects. In the forward condition, a sequence of blocks flashed on the tablet screen, each flash filling the square frame in yellow. Flashing time was set at 500 ms, with an Inter-Onset Interval of 1000 ms). Along with the last block flash, a little blue light turned on in the top-right corner of the screen, informing participants they could start tapping the squares in the same serial order. When tapped, the squares lit up to confirm that the device detected the response. In case of a backward procedure, they were required to tap the blocks in a reverse serial order, from the last block that flashed to the first one. Starting from sequences of two items, if the participants correctly reproduced at least one sequence of the same length, they proceeded to sequences that were one item longer. Participants' span was defined by the last sequence length that the participant repeated with one or no errors before the task was terminated. After assessing the forward and the backward span scores (in a counterbalanced order between subjects), each participant also performed a series of 24 supraspan sequences (procedure adapted from Trojano et al., 1994) in a forward response modality, which was one block longer than the participant's forward span. For each task, the understanding of instructions and tasks was verified with 3 practice trials which were three blocks long.

## Results

For the span we ran a multifactorial ANOVA with gender and age group as between factors and type-of-span (forward vs. backward) as a within-subject factor. Main results showed that the forward span is significantly longer than the backward span, *F*_(1, 103)_ = 21.64, *p* < 0.0001, η^2^ = 0.174 (see Table 1). The between-subject factor age-group was also significant, *F*_(1, 103)_ = 57.83, *p* < 0.0001, η^2^ = 0.360, showing that young adults had longer span than older adults. Gender was also significant, *F*_(1, 103)_ = 5.48, *p* = 0.021, η^2^ = 0.051, showing that male participants had longer span than female participants. The interaction between span and age-group is not significant, *F*_(1, 103)_ = 1.43, *p* = 0.234, η^2^ = 0.014, as well as the interaction between span and gender, *F*_(1, 103)_ = 2.32, *p* = 0.130, η^2^ = 0.022. Interestingly, the three-way interaction between span, age-group and gender is nearly significant, *F*_(1, 103)_ = 3.73, *p* = 0.056, η^2^ = 0.035. The cause of this nearly significant effect is probably due to the fact that women in the older adult group performed much poorer (*M* = 3.81) than men in the same age group and in the same backward condition (*M* = 4.56), and than women in the forward condition (*M* = 4.75).

**Table 1:** **Span and Supraspan scores in all tasks**.

**Measures**								
		**Span FW**		**Span BW**			**Supraspan**	
	***n***	***M***	***SD***	***M***	***SD***	***P***	***PC***	***P***
Younger adults	73	6.109	0.803	5.287	1.199	<0.0001^a^	0.204	0.003
Older adults	34	4.764	1.189	4.352	1.303		0.314	
Female	58	5.559	0.961	4.758	1.368	0.021^b^	0.249	n.s.
Male	49	5.836	1.283	5.265	1.174		0.228	
Younger adults male	32	6.441	0.774	5.516	1.103	0.056^c^	0.178	n.s.
Older adults male	16	4.562	1.1709	4.823	1.199		0.314	
Younger adults female	42	5.857	0.709	5.119	1.238		0.224	
Older adults female	13	4.538	1.082	3.812	1.235		0.315	
All participants	107	5.682	1.132	4.99	1.307	<0.0001	0.239	n.s.

a p-value refers to age-group main effect; interaction between type-of-span and age-group is not significant;

b p-value refers to gender main effect; interaction between type-of-span and gender is not significant;

c *p-value refers to the interaction between type-of-span, age-group, and gender*.

As an alternative measure to the span score, for completeness, we compared the total number of the correctly reproduced sequences in the forward and backward condition for the two age and gender groups. Likewise, span results for score showed that the forward score (*M* = 8.18; *SD* = 2.09) is significantly higher than the backward score (*M* = 6.75; *SD* = 2.31), *F*_(1, 103)_ = 30.84, *p* < 0.0001, η^2^ = 0.230. The between-subject factor age-group was significant, *F*_(1, 103)_ = 43.19, *p* < 0.0001, η^2^ = 0.295 showing that young adults (*M* = 8.95 in the forward score and *M* = 7.27 in the backward score) had higher score than older adult (*M* = 6.37 in the forward span and *M* = 5.53 in the backward span). Gender was also significant, *F*_(1, 103)_ = 4.39, *p* = 0.039, η^2^ = 0.041, showing that male participants (*M* = 8.57 in the forward span and *M* = 7.18 in the backward span) had higher scores than female participants (*M* = 7.84 in the forward score and *M* = 6.38 in the backward score). The two-way interactions between score condition and age-group *F*_(1, 103)_ = 3.44, *p* = 0.066, η^2^ = 0.032 and between score condition and gender, *F*_(1, 103)_ = 1.64, *p* = 0.686, η^2^ = 0.002, are both not significant. The three-way interaction between score condition, age group and gender is not significant either, *F*_(1, 103)_ = 0.68, *p* = 0.410, η^2^ = 0.007.

Regarding the supraspan measure in the forward condition, results indicate that participants were able to correctly perform a significant proportion of trials containing one more item of their formal span forward, in average 24% of all the supraspan trials. Moreover, older adults (*M* = 33% of correct trials) were more correct than younger adults (*M* = 20% of correct trials), *F*_(1, 103)_ = 9.33, *p* = 0.003, η^2^ = 0.083. This data is only apparently in contrast with the superior performance of younger adults in the span tasks, because the supraspan task included, in most cases, more items for younger adults than for older adults and was therefore more difficult. Further evidence of this interpretation is the fact that when a participant scored low in the span task, it was more likely for her/him to score higher in the supraspan task, as demonstrated by the significant negative correlation between the number of items in the supraspan and the proportion of correct trials in the supraspan task, Pearson's *R* = −0.662, *p* < 0.001.

Regarding the temporal features of the task, we measured the delay between the end of the sequence presentation in the screen and the first response tap by the participant (First Tap Latency, FTL, see Table 2). We compared FTLs in the backward and forward conditions and between age and gender groups. Results showed that mean FTL in the forward condition is faster than mean FTL in the backward condition, *F*_(1, 103)_ = 14.70, *p* < 0.001, η^2^ = 0.125. Surprisingly, older adults were faster to react than younger adults, *F*_(1, 103)_ = 22.41, *p* < 0.001, η^2^ = 0.179. While the influence of gender was not significant as a main factor, *F*_(1, 103)_ = 0.58, *p* = 0.446, η^2^ = 0.006, a significant interaction between FTL condition and gender, F *F*_(1, 103)_ = 8.48, *p* < 0.004, η^2^ = 0.051, indicated that male participants were selectively faster than women in the backward condition only, while performing equally in the forward condition. Finally, the three-way interaction between FTL condition, age group and gender was not significant, *F*_(1, 103)_ = 2.81, *p* = 0.096, η^2^ = 0.027.

**Table 2:** **First Tap Latencies in all tasks**.

**Timing measures**									
		**FTL Span FW**		**FTL Span BW**			FTL Supraspan^*^		
	***n***	***M***	***SD***	***M***	***SD***	***P***	***M* correct**	***M* wrong**	***P***
Younger adults	73	1842.67	365.83	1974.31	394.47	<0.001^a^	1717.41	1989.72	0.007^c^
Older adults	34	1509.48	447.25	1719.05	524.46		1528.55	1816.66	
Female	58	1744.93	361.26	1973.57	456.62	<0.004^b^	1722.19	2042.08	0.042^d^
Male	49	1729.10	427.74	1798.08	411.10		1576.30	1807.67	
Younger adults male	32	1826.78	407.81	1896.09	377.09	n.s.	1575.38	1780.36	0.015^e^
Older adults male	16	1603.98	462.10	1629.28	469.67		1577.87	1854.69	
Younger adults female	42	1853.27	334.85	2032.05	407.29		1825.79	2144.24	
Older adults female	13	1403.17	430.58	1820.05	586.11		1476.14	1773.88	
All participants	107	1737.39	391.70	1893.20	435.77	<0.001	1655.08	1934.73	<0.001

* For all analyses in this column n = 100, please see text for details;

a p-value refers to age-group main effect;

b p-value refers to the condition and gender interaction;

c p-value refers to age-group main effect;

d p-value refers to gender main effect;

e *p-value refers to age-group and gender interaction*.

Another interesting temporal parameter of the performance is the mean intertapping interval (ITI), which can be used as a measure of tapping pace. Results showed that participants tapped the screen with a frequency of about 600 ms. Mean ITIs in the forward condition (*M* = 593 ms, *SD* = 258) and in the backward condition (*M* = 639 ms, *SD* = 258) were not significantly different, *F*_(1, 103)_ = 0.773, *p* = 0.381, η^2^ = 0.007. Also, no effects of age group, *F*_(1, 103)_ = 0.21, *p* = 0.885, η^2^ = 0.000, gender *F*_(1, 103)_ = 0.992, *p* = 0.321, η^2^ = 0.010, and no interactions were found.

Finally, we compared FTLs in the wrongly and correctly recalled sequences in the supraspan condition for the two age and gender groups (see Table 2). Seven participants were excluded from the analysis because they did not perform correctly any of the 24 sequences of the supraspan condition. Results showed that mean FTL for the correctly recalled sequences was faster than mean FTL for the wrongly recalled sequences, *F*_(1, 96)_ = 48.80, *p* < 0.001, η^2^ = 0.337.

Confirming the surprising results of the span task, older adults were faster to react than younger adults, *F*_(1, 96)_ = 7.58, *p* = 0.007, η^2^ = 0.073. The influence of gender was also significant, *F*_(1, 96)_ = 4.22, *p* = 0.042, η^2^ = 0.042, with male participants being faster than female ones. Interestingly, a significant interaction between age group and gender indicated that male participants were selectively faster than women in the younger group, but not in the older group, *F*_(1, 96)_ = 6.07, *p* = 0.015, η^2^ = 0.070. All the other interactions between factors were not significant.

## Discussion

We tested eCorsi with a battery of spatial working memory tests: span forward, span backward, and supraspan forward. Our results were not substantially different from previous CBTT normative data about the span (Smyth and Scholey, 1994; Kessels et al., 2000, 2008; Nelson et al., 2000; Vandierendonck et al., 2004), showing that our digitized version of the test worked similarly to the traditional version. The absence of substantial differences leads to the conclusion that eCorsi could be safely used to substitute the traditional CBTT in clinical and research practices.

Our main results show an advantage of the forward condition in terms of higher span, greater absolute number of correctly recalled sequences and faster First Tapping Latency (FTL) in correct sequences. These findings are consistent with Vandierendonck et al. (2004), who show a significant advantage of the forward condition over the backward one and in contrast with Kessels et al. (2008) and Cornoldi and Mammarella (2008), which showed no difference in accuracy for the forward and backward conditions. Following the interpretation of Vandierendock and colleagues, we explain the forward condition advantage as the consequence of a different involvement of the working memory subsystems in the two tasks. While the forward condition appears to rely mainly on the visuospatial sketchpad, involving the central executive only with longer sequences, the backward condition puts a heavier load on the central executive (e.g., to reverse the sequence). This explanation is also supported by a faster response in the forward than in the backward condition (see below for a discussion about timing data).

Consistently with previous studies, (Orsini et al., 1987; Kessels et al., 2000), aging affected both forward and backward spans. Concerning gender differences, analogously with previous findings (Grossi et al., 1980; Orsini et al., 1987, but see also Kessels et al., 2000 for contrasting results), we found that males performed a little better than females. The reason of this gender difference is likely due to a general advantage of male over female participants on visuospatial tasks. Such superiority is well documented in literature (e.g., Lewin et al., 2001), and it seems to be grounded in different visuospatial processes (Weiss et al., 2003).

Concerning the supraspan results, we found that most of participants could correctly recall a certain number of sequences exceeding their own span. This result confirms that the span is not an absolute memory threshold that cannot be overpassed and that supraspan measures are useful to explore performance limits. Supraspan is also useful to investigate incidental learning curves, applying Hebb's (1961) method. In addition, being based on a series of sequences of the same length, it offers the opportunity for personalized and clearer error and ITI analyses. However, it is worth noting that the supraspan measure we used (Trojano et al., 1994) is not an absolute measure, as the length of the sequence varies according to the subject's span. Consequently, it always has to be considered in association with the span performances, since participants with longer span are more prone to errors in the supraspan. When comparing different groups, such negative correlation between span and supraspan leads to apparently odd results, like older adults outperforming younger adults in the supraspan condition while being clearly outperformed by the younger group in the span procedure.

Concerning the temporal aspects of the task, we found that the FTL in the forward span condition is shorter than the one in the backward condition. This result may appear counter-intuitive, because in the backward condition the subject has just encoded the block to be tapped first. We explain this temporal advantage of forward reproduction with the fact that the entire sequence should be planned before starting the execution. While in the forward condition participants can start planning the response sequence as soon as the sequence starts and update with new blocks to the sequence online, in the backward condition, they must wait for the end of the sequence to start planning the response. This idea is consistent with the hypothesis of a heavier load on the central executive in the backward condition proposed by Vandierendonck et al. (2004) (see the above discussion about forward and backward span differences).

Similar timing results were recently reviewed by Hurlstone et al. (2014): using a verbal span task, in the case of backward recall, participants leave a pause before starting with the first item (that is in this case the last item in the sequence). The authors assume that recall of the first item in the backward task is delayed due to the time to program the sequence in a reversed order (p. 0.5). This idea is also confirmed by Haberlandt et al. (2005) using a verbal span task: they suggest that the output of the first item coincides with the planning of the entire sequence and this slows down the response at the first position, the *multiple-scan strategy* (Conrad, 1965; Murdock, 1995). Similar results were also obtained by Thomas et al. (2003): in this case the authors suggest that a backward request in a verbal span task involves implicit multiple scan to the beginning of the list, thus participants reverse the order of items by scanning back to the first input item and then advance to the current target item. Since our pattern of results mirrors quite accurately those obtained with verbal tasks, we can speculate that the strategy to recall a series of locations in a backward order is rather similar to the one used for verbal materials. Moreover, given the nature of the operations involved in such a task (e.g., scanning, reversing), we reckon that this may be additional evidence of the enhanced involvement of the central executive.

To conclude, our data, in line with recent literature, show that the interval between the sequence end and the response is a crucial processing interval. This assumption is also indirectly confirmed by Fischer (2001), who finds that movement initiation time (FTL in our terms) increases nonlinearly with sequence length, making it measure sensitive (and therefore probably connected) to spatial memory limits.

Moreover, the difference between FTLs for correct and wrong sequences seems to point in the same direction. When about to perform a wrong forward sequence, participants show a significantly longer FTL: this result is consistent with the idea that the interval between the sequence end and the beginning of response is a crucial moment that can be used as a predictor of success-failure and processing load. All of these time-related results taken together, point out, confirming and expanding Fischer's (2001) conclusions, that this time interval is particularly sensitive to memory processing and misprocessing, possibly indicating central executive involvement.

Visuospatial impairment is linked to diseases like Parkinson, Alzheimer (Ala et al., 2001; Cormack et al., 2004), schizophrenia, attention deficit hyperactivity disorder, and traumatic brain injury (Gagnon and Belleville, 2011; Gorman et al., 2012). Traditionally, the diagnosis of cognitive abilities in these deseases relies on neuropsychological tests (Petersen, 2004). Many studies show that these patients perform worse than healty subjects of similar age at WM tests (Brown and Marsden, 1991; Trojano et al., 1994; Tiraboschi et al., 2006; Saka and Elibol, 2009). Moreover, in a recent work by Mammarella and Cornoldi (2005), it is shown that children with disabilities perform worse in a backward CBTT. The eCorsi expands the potential of traditional CBTT with accurate timing measurement, allowing, for example, an early diagnosis of some neurological deficits that involve impaired spatial abilities. For instance, since there is evidence that the central executive WM subsystem is impaired in Parkinson's patients (Dalrymple-Alford et al., 1994), following the claim that a crucial part of subjects response is planned in the pause between the end of the presentation and the beginning of the response (along with the relative involvement of the central executive), with eCorsi it would be possibile to investigate the performance of this kind of patients using FTL durations. Similarly, FTL could be a sensitive measure in the diagnostic process of all the conditions listed above, as well as a tool for measuring treatment efficacy.

## Limitations

Some of the results we obtained (e.g., older adults being faster than younger adults) could be due to the difference in sample numerosity (34 and 73): a *t*-test of the FTL values for the older and younger groups showed that the *SD*s are statistically different. As *SD* diminishes as a sample becomes bigger, we cannot exclude that the difference between the two samples is due also to the size of the samples themselves. While not being conclusive, this additional analysis confirms that the two groups feature different variabilities: given that the two groups are numerically different, it is thus unclear if the FTL difference between younger and older adults is due to numerosity and/or age.

Furthermore, another possible limitation of this research could be the use of the supraspan measure (Trojano et al., 1994) that, while being more sensitive and offering more information than the classic span procedure, it is by definition different between participants (e.g., being dependent on the individual span score). For example, this may generate specific issues when directly comparing response latencies between participants, as shorter sequences are more likely to generate shorter reaction times.

## Conclusions

In this paper we described the features of eCorsi, a digitized version of the CBBT. In summary, eCorsi, is easy to install, customize and use, and allows an intuitive screening of participants' results. When compared to traditional CBBT, eCorsi shows several advantages, including an increased accuracy in the presentation timing, an automatic measure of span and reaction times, the possibility of fully customized sequences, the implementation of a monitor function which enables the tester to remotely follow subjects' performances, and many other minor features. Also, compared to other digitized versions of the CBBT, eCorsi seems to be the most complete and versatile one. We tested a total of 107 participants with different Corsi tasks implemented in eCorsi. Results showed that span and error rates were essentially analogous to the ones obtained in the main standardization studies which have used the original physical version of CBBT. Finally, results about the temporal features of span performance provide new insights about the mechanisms underlying spatial sequence processing, expanding theories about the involvement of working memory components in performing CBTT and open the way to further investigations. In particular, our data suggest that subjects' response is not planned during sequence presentation, but in the time interval between the end of the presentation and the beginning of subjects' response.

All the features we described show that eCorsi is not only useful for diagnostic, but can be very useful in the field of research, as it allows the analysis of spatial working memory mechanisms otherwise difficult, and sometimes impossible to be investigated with traditional methods (e.g., temporal aspects both in presentation and in response phases, variables isolation, log file analysis for error studies, etc.). Moreover, we believe that eCorsi is more accessible and user-friendly than other automated or digitized CBTT versions, as it is based on standardized technology (tablets) which is nowadays widely available.

### Conflict of interest statement

The authors declare that the research was conducted in the absence of any commercial or financial relationships that could be construed as a potential conflict of interest.

## Footnotes

^1^The educational level measured in years of schooling for the two age groups was: young average 15.07, *SD* = 1.73; old average 14.29, *SD* = 2.66. A *t*-test between the two distributions obtained a non-significant *p*-value of 0.127.

^2^Recently we developed an additional version of the program, where “Touch OSC” is replaced by “MIRA,” a controller specifically designed for the iPad by Cycling'74. For the purposes of this study, however, we will always be referring to the “TouchOSC” version.
