# Supplementary material for: eCorsi: implementation and testing of the Corsi block-tapping task for digital tablets
Source: Front Psychol. 2014 Sep 2;5:939. doi: 10.3389/fpsyg.2014.00939 (PMC4151195; doi:10.3389/fpsyg.2014.00939)
Supplement: Supplementary file 1 [file DataSheet1.DOCX]

**Appendix**

**Technical specifications**

*ADAPTATION TO THE IPAD SCREEN*

In order to adapt the Corsi block-tapping test to the iPad screen, all coordinates were multiplied by 4 (and thus read in pixels) from the ones expressed in millimeters in the study by Kessels et al., (2000), thus obtaining a total area of 1020(hor.) x 820(vert.). Considering that each block thus becomes a 120x120 pixels square (23mm per side), and that the coordinates indicate here upper leftmost corner of each square (the 0,0 origin is in the upper leftmost corner of the screen area: X goes rightwards, while Y goes downwards); the resulting coordinates for each block are (X, Y):

1: 520, 80

2: 120, 120

3: 720, 210

4: 280, 260

5: 560, 340

6: 780, 460

7: 60, 500

8: 300, 620

9: 540, 580

To center it on the iPad using touchOSC (available area 1024 x 728), however, since the horizontal axis is slightly smaller than the touchOSC available layout screen (2 pixels shorter), and the vertical axis is slightly longer than the layout screen (92 pixels larger). We thus added 1 pixel to each X coordinate (half of the correction needed, to center it on the screen) and we subtracted 46 from each Y coordinate.

Lastly, a general shift towards the right of 30 pixels was useful to center the whole structure to the available screen area. Notice that all these adjustments did not affect the spatial relationships between the blocks, which remain unaltered.

Thus, Block 1 resulted in position (551, 34), Block 2 (151, 74), etc. in touchOSC editor. Finally, we turned the whole structure upside-down, in order to simulate the original subject’s view on the iPad screen. Final block coordinates are as follows (squares’ uppermost left corner):

1: 353, 574

2: 753, 534

3: 153, 444

4: 603, 394

5: 313, 314

6: 103, 194

7: 813, 154

8: 573, 34

9: 333, 74

*SPAN SEQUENCES*

The two sequences are taken, for the forward and backward versions respectively, from the standardizations by Kessels et al. (2000) and Kessels et al. (2008). The sequences above length 8 were created according to criteria similar to those used by Kessels and colleagues.

Forward sequences

Length 2

4 7

2 9

Length 3

9 3 4

6 3 7

Length 4

1 5 2 8

7 4 3 9

Length 5

3 1 8 6 5

9 3 1 4 7

Length 6

2 8 3 5 6 4

5 3 1 2 8 9

Length 7

7 3 2 9 1 8 6

4 3 7 6 2 5 9

Length 8

1 9 6 3 5 4 2 8

2 9 4 6 1 7 3 5

Length 9

5 3 8 7 1 2 4 6 9

4 2 6 8 1 7 9 3 5

Length 10

9 8 5 2 3 1 6 7 4 3

1 8 2 4 6 7 1 3 9 5

Length 11

5 2 8 3 1 7 6 9 4 7 8

4 2 7 1 5 6 8 3 9 4 3

Backward sequences

Length 2

8 5

6 4

Length 3

4 7 2

8 1 5

Length 4

3 4 1 7

6 1 5 8

Length 5

5 2 1 8 6

4 2 7 3 1

Length 6

3 9 2 4 8 7

3 7 8 2 9 4

Length 7

5 9 1 7 4 2 8

5 7 9 2 8 4 6

Length 8

5 8 1 9 2 6 4 7

5 9 3 6 7 2 4 3

Length 9

5 3 8 7 1 2 4 6 9

4 2 6 8 1 7 9 3 5

Length 10

9 8 5 2 3 1 6 7 4 3

1 8 2 4 6 7 1 3 9 5

Length 11

5 2 8 3 1 7 6 9 4 7 8

4 2 7 1 5 6 8 3 9 4 3
